# Continuous Positive-Pressure Oxygen Supply Improves Fiberoptic Intubation Efficacy and Safety in Patients With Simulated Cervical Spinal Injury: A Prospective Double-Blind Randomized Controlled Study

**DOI:** 10.7759/cureus.66802

**Published:** 2024-08-13

**Authors:** Shi Qinye, Zhu Tianlun, Zhou Youfa, Chen Gang

**Affiliations:** 1 Anesthesia, Zhejiang University School of Medicine Sir Run Run Shaw Hospital, Hangzhou, CHN

**Keywords:** anesthesia induction, cervical spine injury, oxygen, intubation, fiberoptic

## Abstract

Background: Fiberoptic intubation is an important method for tracheal intubation in patients with cervical spine injury. How to effectively and safely complete fiberoptic intubation while maintaining the stability of the cervical spine is very important. This study compared the efficiency and safety of fiberoptic intubation after anesthesia induction under different types of air pressure in patients with simulated cervical spinal injury.

Methods: In total, 59 adult patients who underwent fiberoptic intubation with a cervical collar for simulated cervical spinal injury were randomly allocated to continuous positive-pressure oxygen, normal-pressure, or intermittent negative-pressure suction groups. After the induction of anesthesia and adequate 100% oxygenation, which confirmed effective neuromuscular blockade, it was deemed appropriate to begin fiberoptic intubation. In the continuous positive-pressure oxygen group, the fiberoptic device was connected through the negative-pressure suction path with 5 L/min oxygen. In the intermittent negative-pressure suction group, the fiberoptic device was connected to the negative-pressure suction device. In the normal-pressure group, the flexible fiberoptic device was not connected to either the oxygen source or the negative-pressure suction device. The intubation time was recorded as the primary outcome measure. The intubation success rate, number of attempts, minimum SpO2, objective lens contamination rate, and incidence of complications were also compared among the groups.

Results: Compared with those in the other groups, the median (range) intubation time in the continuous positive-pressure group was 59 (36-181) seconds, which was significantly shorter than that in the normal-pressure group, 167 (46-181) seconds, and the intermittent negative-pressure suction group, 132.5 (38-181) seconds (P=0.04). The success rate of nasotracheal intubation was significantly greater in the continuous positive-pressure group (94.7%, 18/19) than in the normal-pressure group (50%, 10/20) and intermittent negative-pressure suction group (50%, 10/20) (P=0.004). There was a significant difference among the three groups (P=0.043). The median (range) minimum SpO2 during fiberoptic intubation was 100% (99-100%) in the continuous positive-pressure group, 100% (90-100%) in the normal-pressure group, and 99% (88-100%) in the intermittent negative-pressure suction group (P=0.029). However, no statistically significant difference was detected among the groups with complications.

Conclusion: The continuous use of positive-pressure oxygen via the negative-pressure suction pathway can improve the efficiency and safety of fiberoptic intubation in patients with simulated cervical spinal injury after anesthesia induction.

## Introduction

In recent years, the number of cervical spine fractures has gradually increased [1]. Spinal cord injury caused by cervical spine fractures usually requires tracheal intubation for mechanical ventilation [2]. Anesthesiologists should ensure airway safety and avoid iatrogenic nerve injury during tracheal intubation in patients with cervical spine fracture [3]. The use of a cervical collar is a common nursing method for stabilizing cervical spine fractures [3]. However, the cervical collar limits cervical mobility, mouth opening, and jaw lifting.

In patients with predictable difficult airways such as cervical spine fractures, anesthesiologists usually perform awake intubation. However, mild cough during awake intubation is unavoidable [4]. After anesthesia induction, fiberoptic nasotracheal intubation cannot be affected by the cervical collar and can avoid the movement of the cervical spine caused by coughing during awake intubation.

Fiberoptic intubation is important for managing a difficult airway [5,6], but fiberoptic intubation is a technique that requires considerable skill and practice [7,8]. In patients with cervical spine fracture, the tongue falls back after anesthesia induction, which reduces the oral volume and increases the difficulty of fiberoptic intubation. At the same time, it is difficult to increase the oral volume and reduce the difficulty of intubation by cervical mobility and jaw lifting after neck fixation.

Often, hypoxia and multiple attempts are required to complete endotracheal intubation during awake fiberoptic intubation [4,9], reducing the safety and efficiency of the procedure. Intermittent negative-pressure suction (INS) is the traditional nonpharmaceutical method for assisting fiberoptic intubation, but it creates negative pressure in the oropharyngeal cavity.

Oxygenation is very important in difficult airway management and fiberoptic intubation [10]. Different forms of oxygen administered during fiberoptic intubation can reduce the incidence of hypoxia during intubation [11-14]; thus, this approach can improve the safety of fiberoptic intubation. Roh et al. reported that oxygen insufflation through the working channel of the fiberoptic can help in extending the apneic window after the induction of general anesthesia during fiberoptic intubation [15]. This method can produce continuous positive-pressure oxygen (CPO) in the oropharynx. However, whether this nonpharmacological method is more suitable than other nonpharmacological methods for fiberoptic intubation under many conditions of limited intubation, such as cervical spine fracture, is unclear.

This prospective randomized controlled study compared the efficacy and safety of different pressures (continuous positive pressure, normal pressure, and intermittent negative pressure) on fiberoptic nasotracheal intubation in simulated cervical spine fracture patients (a cervical collar was used to limit the movement of the cervical spine) after anesthesia induction.

## Materials and methods

The study was approved by the Ethics Committee of the Fourth Affiliated Hospital of Zhejiang University Medical College (approval number: k2021092). As inclusion criteria, 60 included subjects were patients aged 18-65 years who needed oral surgery requiring nasotracheal intubation and were evaluated as American Society of Anesthesiologists (ASA) grades I-II from September 2021 to December 2023. The exclusion criteria were skull base fracture, abnormal coagulation function, nasal atresia or nasal stenosis, nasal bone fracture, other contraindications to nasotracheal intubation, and refusal to undergo fiberoptic nasal intubation. All patients were immobilized with a neck brace to limit cervical spine movement to simulate the protective effect of a neck brace for cervical spine fractures. We recorded the patients' age, sex, weight, height, body mass index (BMI), ASA physical status, and Mallampati grade.

The trial was registered prior to patient enrollment at the Chinese Clinical Trial Registry (registration number: ChiCTR2100051439). Also, the manuscript was approved under the International Committee of Medical Journal Editors (ICMJE) guidelines.

Randomization and grouping

The subjects were randomly divided into a CPO group, a normal-pressure (NS) group, and an INS group at a distribution ratio of 1:1:1. The patients were grouped according to a computer-generated random number list, and information about the grouping was sealed in an envelope that was handed by a professional nurse to the experimenter before anesthesia induction.

In the CPO group, the flexible fiberoptic device was connected through a negative-pressure suction path to the oxygen source. During flexible fiberoptic guidance, 5 L/min of oxygen was drawn into the airway, and oxygen was supplied continuously under positive pressure.

In the INS group, the flexible fiberoptic device was connected through a negative-pressure suction path to the negative-pressure suction device. Intermittent negative-pressure suction was applied to remove secretions if any were found to affect the visual field during intubation.

In the NS group, the flexible fiberoptic device was not connected to either the oxygen source or the negative-pressure suction device.

Anesthesia and fiberoptic intubation

Before the operation, the patient was asked about the smoothness of nasal airflow on both sides, and the side with the smoothest airflow was selected for nasotracheal intubation, with the opposite side used for standing. After the operation began, electrocardiogram (ECG), blood pressure, and SpO2 were monitored. Anesthesia was induced with 1.5 mg/kg lidocaine, 1.2 mg/kg propofol, 0.5 µg/kg sufentanil, and 0.6 mg/kg rocuronium. After the induction of anesthesia and adequate 100% oxygenation, which confirmed effective neuromuscular blockade, it was deemed appropriate to begin intubation. Fiberoptic intubation was performed using a flexible electronic fiberoptic device (Zhejiang Youyi Medical Instrument Co., Ltd., China, model TIC-SD-III). The flexible electronic fiberoptic was 600 mm in length, 4.5 mm in outer diameter, and 2.2 mm in inner diameter (i.e., the diameter of the duct) and was equipped with a video recording function.

This experiment was performed by the same anesthetist who had performed more fiberoptic nasotracheal intubations [16]. After inducing general anesthesia and pre-oxygen, the experimenter selected a method of fiberoptic intubation according to the grouping information and recorded a video of the operation process through the built-in video function of the fiberoptic. Once tracheal intubation was accomplished, confirmation of the position of the endotracheal tube was accomplished by capnography. After intubation, the video was given to another anesthesiologist (the result analyst), who was unaware of the experimental procedure, to record the results.

Outcome variables

We simulated a patient with a cervical spine fracture. To ensure the safety of the simulated patients, we had to complete the intubation within 180 seconds, the number of intubation attempts was not more than 4, and the SPO2 was not lower than 93%; otherwise, tracheal intubation was considered to have failed [16]. If intubation was unsuccessful, the anesthesia assistant released the cervical collar and manipulated the mandible to expose the glottis. In such cases, the intubation time was 181 seconds, and one case of intubation failure occurred. If manipulation of the mandible by the assistant still failed to effectively expose the glottis, the bronchoscope was withdrawn to allow oxygen supply with a pressurized mask, and the videolaryngoscope was replaced for tracheal intubation or combined with the bronchoscope to complete tracheal intubation [17].

We recorded the intubation time and number of attempts. The intubation time was defined as the time from the insertion of the bronchoscope in the nostril to the completion of tracheal intubation; this duration did not exceed 180 seconds. In some cases, the anatomical structure of the throat could not be effectively identified under the bronchoscope, or the objective lens of the bronchoscope was contaminated by secretions. If necessary, the bronchoscope was withdrawn and repositioned or cleared of secretions as necessary to provide a satisfactory anatomical view. Each insertion of the bronchoscope was considered an attempt; the number of attempts was less than 4.

The minimum SpO2 was recorded. The minimum SpO2 during the intubation process was defined as the minimum SpO2 in the period from the establishment of an oxygen supply via the pressurized mask to the fully intubated patient being connected to the ventilator.

The occurrence of visual field obstruction caused by contamination of the objective lens and complications of intubation were also compared among the groups. Contamination of the objective lens was defined by the presence of oral secretions or blood on the lens that resulted in an unclear field of view. Once the patients were intubated, they were connected to a ventilator and mechanically ventilated. Any complications during intubation were recorded.

Sample size calculations and data analysis

In this preliminary experiment, the sample size was calculated according to the results of flexible fiberoptic nasotracheal intubation: the required durations were 52.2±11.5 s, 105±54.3 s, and 155±80 s in the CPO group, NS group, and INS group, respectively. The CPO group was compared with the NS group, yielding α=0.05 and β=0.08. Given that the calculated significant difference was the maximum sample size, the required sample size for both groups was 17 patients each. With an estimated dropout rate of 20%, the sample size of each group was expected to be 20 patients, and the total number of patients was expected to be 60.

Statistical analyses were performed using IBM SPSS Statistics for Windows, Version 23.0 (Released 2015; IBM Corp., Armonk, New York, United States). The distributions of continuous variables were analyzed for normality using the Shapiro-Wilk test. Qualitative data are described as numbers and percentages (%). The data are presented as the number or the mean±SD for variables with a normal distribution or as the median (range) for variables with a nonnormal distribution. Normally distributed data were compared using repeated-measures multivariate analysis of variance (ANOVA). Nonnormally distributed data were compared by the Kruskal-Wallis test, and the Bonferroni correction was used to adjust significance values for multiple group comparisons. Categorical data are presented as numbers and percentages (%). Fisher's exact test or the χ2 test was used to analyze categorical data. All comparisons were two-sided, and a p-value less than 0.05 was considered to indicate statistical significance.

## Results

**Figure 1:**
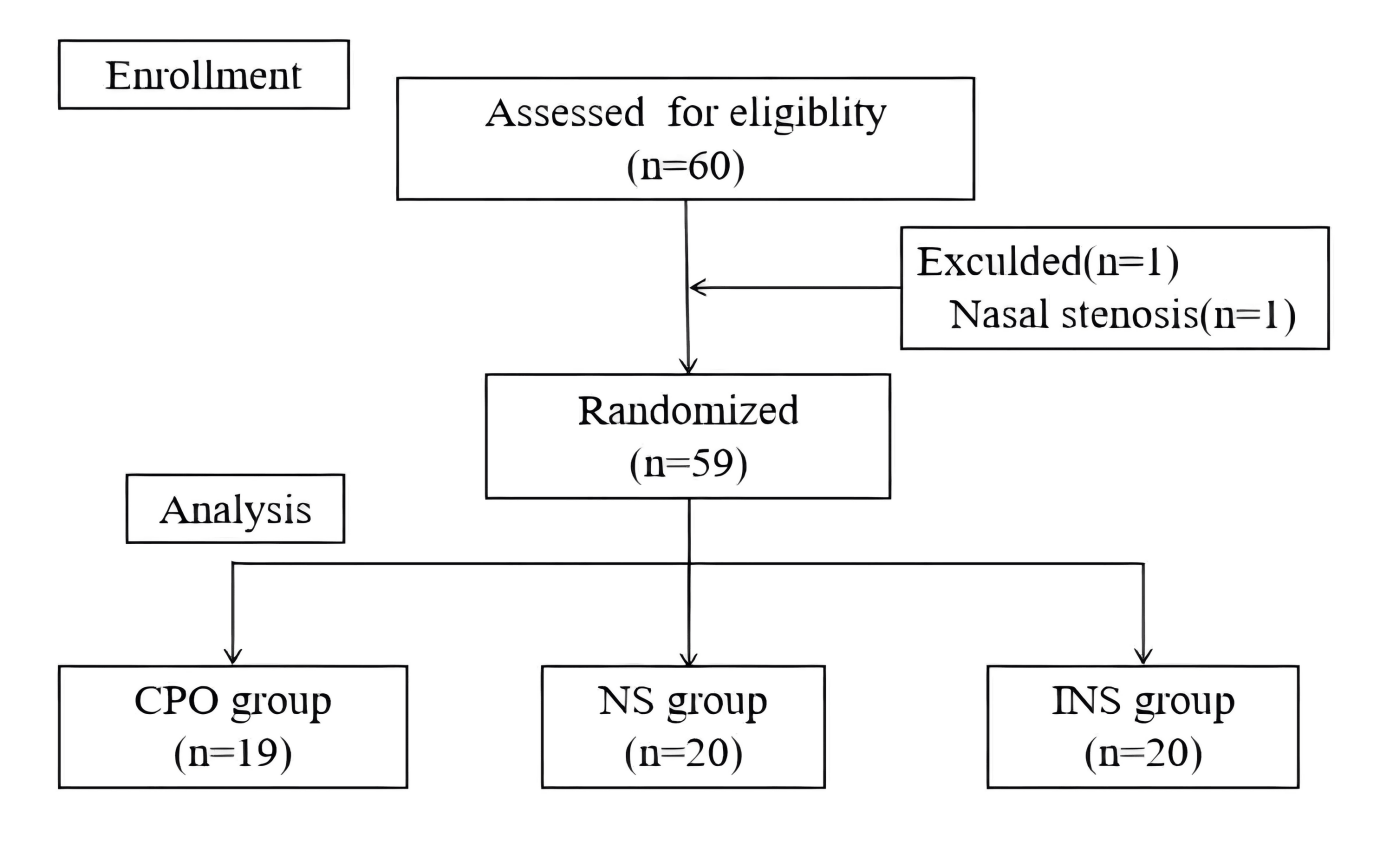
CONSORT diagram showing the flow of participants CONSORT: Consolidated Standards of Reporting Trials; CPO: continuous positive-pressure oxygen; NS: normal-pressure; INS: intermittent negative-pressure suction

In total, 60 patients were randomly allocated to each group. One patient was excluded because the endotracheal tube could not ventilate the nasal cavity because of nasal stenosis after successful fiberoptic guidance, and intubation was successful after the replacement of a thinner endotracheal tube (Figure 1).

Data from 59 subjects were analyzed. The demographic and clinical characteristics of the patients are shown in Table 1. There were no differences in patient characteristics among the three groups (Table 1).

**Table 1:** Demographic and clinical characteristics of the patients Data are shown as the mean±SD or number (proportion). SD: standard deviation; ASA: American Society of Anesthesiologists; BMI: body mass index; CPO: continuous positive-pressure oxygen; NS: normal-pressure; INS: intermittent negative-pressure suction

	CPO (n=19)	NS (n=20)	INS (n=20)	P-value
Age, mean years±SD	40.9±11.3	31.7±11.7	37.4±13.6	0.067
Sex, n (%)				0.209
Male	12 (63.6)	13 (65)	8 (40)	_
Female	9 (47.7)	7 (35)	12 (60)	_
Height (cm), mean±SD	163.7±7.11	167.6±8.5	165.2±7.5	0.263
Weight (kg), mean±SD	62.3±12.1	60.1±12.4	62.7±9.5	0.953
BMI (kg/m^2^), mean±SD	23.3±12.3	21.6±3.7	22.9±3.5	0.318
ASA physical status, n (%)				0.33
I	3 (15.8)	7 (35)	4 (20)	_
II	16 (84.2)	13 (65)	16 (80)	_
Mallampati grade, n (%)				0.268
I	1 (5.3)	4 (20)	5 (25）	_
II	6 (31.6)	10 (50)	8 (40）	_
III	9 (47.4)	3 (15)	4 (20)	_
IV	3 (15.8)	3 (15)	3 (15)	_

**Figure 2:**
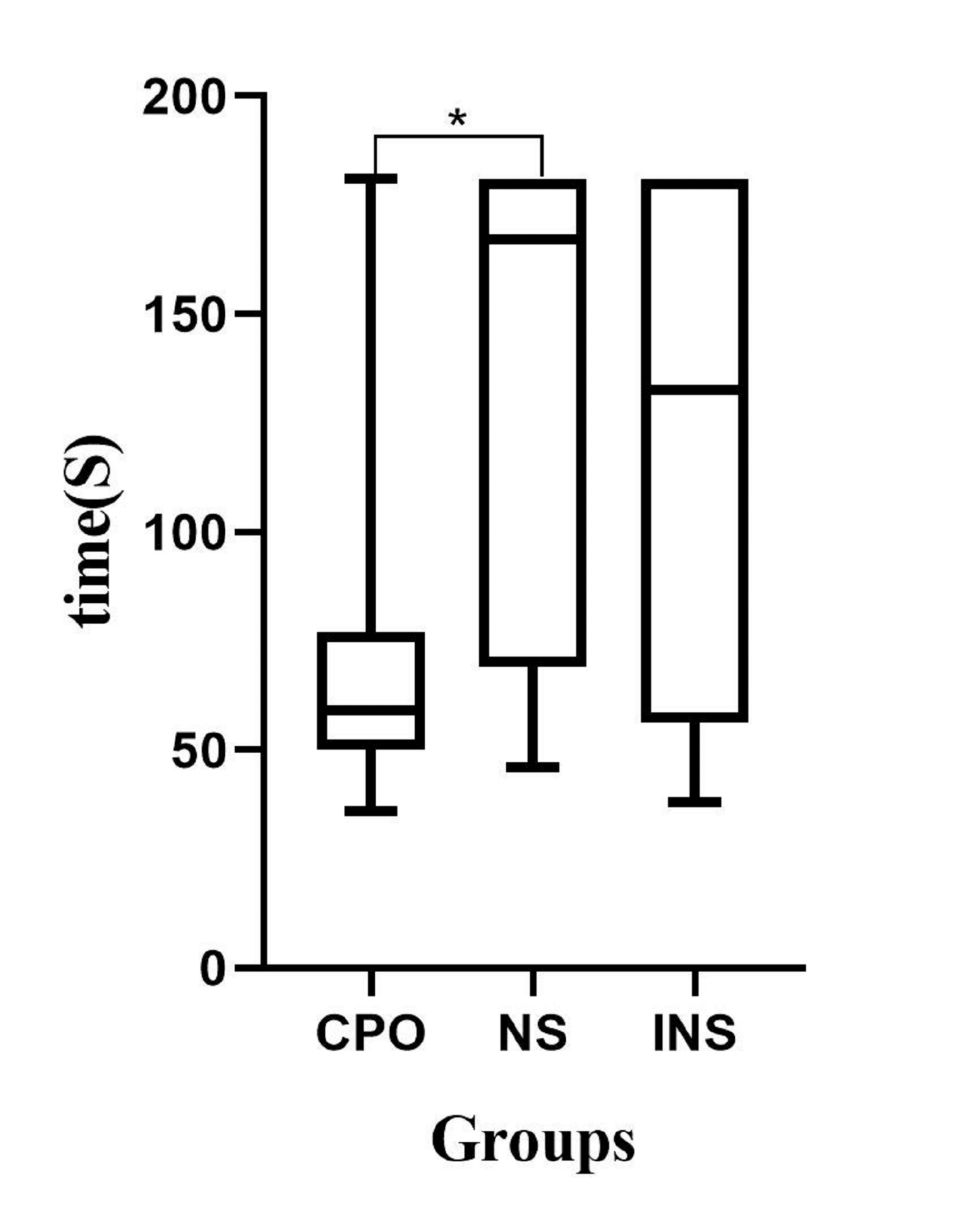
Comparison of the intubation time among the groups *P<0.05 compared with the two other groups. CPO: continuous positive-pressure oxygen; NS: normal-pressure; INS: intermittent negative-pressure suction

The median (range) intubation time in the CPO group, which was 59 (36-181) seconds, was shorter than that in the NS group, which was 167 (46-181) seconds, and in the INS group, which was 132.5 (38-181) seconds (P=0.04). There were significant differences between CPO and NS (P=0.005). However, there was no significant difference between CPO and INS (P=0.051) or between NS and INS (P=1) (Figure 2).

The success rate of nasotracheal intubation was significantly greater in the CPO group (94.7%, 18/19) than in the NS group (50%, 10/20) and INS group (50%, 10/20) (P=0.004). There was a significant difference between the CPO and NS groups (P=0.002) and between the CPO and INS groups (P=0.002). However, there was no significant difference between the NS and INS groups (P=1) (Table 2).

**Table 2:** Comparison among studied groups according to intubation success rate, attempts, SPO2, objective lens contamination, and complications Data are shown as the mean±SD, or number (proportion), or 95% CI. Statistically significant p-values are highlighted in bold. SD: standard deviation; CI: confidence interval; SPO2: pulse oxygen saturation; CPO: continuous positive-pressure oxygen; NS: normal-pressure; INS: intermittent negative-pressure suction

	CPO (n=19)	NS (n=20)	INS (n=20)	P-value
Success rate of intubation, n (%)	18 (95)	10 (50)	10 (50)	0.004
SPO2, 95% CI	99.5-100.0	97.8-100.0	96.0-99.5	0.029
Number of attempts, n (%)				0.027
0	10	4	7	
1	5	3	1	
2	3	1	1	
3	0	4	2	
4	1	8	9	
Objective lens contamination, n (%)	0 (0)	6 (30)	7 (35)	<0.001
Complications, n (%)	0 (0)	0 (0)	1 (5)	0.371

The number of attempts was analyzed according to grade. There was a significant difference among the CPO, NS, and INS groups (P=0.043) (Table 2). There were significant differences between the CPO and NS groups (P=0.026) and between the CPO and INS groups (P=0.014). There was no significant difference between the NS and INS groups (P=0.787) (Table 2).

The median (range) minimum SpO2 during fiberoptic intubation was 100% (99-100%) in the CPO group, 100% (90-100%) in the NS group, and 99% (88-100%) in the INS group. There were significant differences in the overall distribution among the three groups (P=0.029) (Table 2). However, there was no significant difference between the CPO and NS groups (P=1), between the CPO and INS groups (P=0.068), or between the NS and INS groups (P=0.062).

The objective lens contamination rate was 0% (0/19) in the CPO group, 30% (6/20) in the NS group, and 35% (7/20) in the INS group, indicating a significant difference (P<0.005) (Table 2). One patient in the INS group was found to have scattered bleeding points in the oral mucosa caused by negative-pressure suction during flexible fiberoptic guidance (P=0.371). The minimum SpO2 was 93% in three of the 20 patients in the INS group and in one of the 20 patients in the NS group, and the minimum intubation duration exceeded 180 seconds in one of the 20 patients in the NS group. The glottis failed to be effectively exposed after three attempts in one of the 19 patients in the CPO group, eight of the 20 patients in the NS group, and seven of the 20 patients in the INS group, in whom intubation was completed with the assistance of lifting the mandible (Table 3).

**Table 3:** The glottis failed to be effectively exposed Data are shown as the number (proportion). Statistically significant p-values are highlighted in bold. SPO2: pulse oxygen saturation; CPO: continuous positive-pressure oxygen; NS: normal-pressure; INS: intermittent negative-pressure suction

	CPO (n=19)	NS (n=20)	INS (n=20)	P-value
The glottis exposed failure				0.026
Minimum SPO2 ≤93%, n (%)	0 (0)	1 (5)	3 (15)	
Intubation time ≥180 s, n (%)	0 (0)	1 (5)	0 (0)	
Number of attempts ≥4, n (%)	1 (5.3)	8 (40)	7 (35)	

## Discussion

Anesthesiologists should avoid iatrogenic nerve injury caused by cervical spine movement caused by tracheal intubation in patients with cervical spine fracture. Fiberoptic intubation has the least impact on cervical spine movement [18]. However, it is more difficult for anesthesiologists to perform tracheal intubation in patients with cervical spine fractures after anesthesia induction and limit cervical spine movement. Therefore, we simulated the nasotracheal intubation of a cervical spine fracture patient by immobilizing the patient with a cervical collar while the patient was fiberoptic intubated after general anesthesia induction.

CPO administration through the negative-pressure suction pathway can assist anesthesiologists in performing fiberoptic nasal intubation as efficiently and safely as possible after general anesthesia induction under conditions of limited cervical spine movement by a cervical collar. In the case of cervical spine inactivity, continuous-positive pressure oxygen administration-assisted tracheal intubation with fiberoptic bronchoscopy has more advantages.

At the same time, to ensure the safety of the procedure for simulated patients, we propose that the operator must have the following requirements: the intubation time must be less than 180 seconds, the number of intubations must be less than 4, and the blood oxygen saturation during intubation must not be less than 93% [4]. Therefore, the success rate of fiberoptic intubation in the control group (50%) in this study was lower than that according to the expert consensus (78-100%) [6]. However, under the conditions of high operational requirements and complete cervical immobilization, CPO can improve the success rate of fiberoptic intubation (95%), which is similar to the findings of expert consensus. Therefore, CPO can improve the success rate of fiberoptic intubation.

Haghshenas [19] reported a median awake flexible intubation time of 80 s in predictable difficult airway patients. Kramer et al. [20] reported that the median time required for flexible intubation in patients with cervical spine injury was 102 s. All the above studies were performed during awake tracheal intubation. As a result of general anesthesia induction, gravity and relaxation of the upper respiratory muscles lead to the collapse of the tongue root and soft palate [21], reducing the volume of the pharyngeal cavity. Accordingly, fiberoptic intubation is more difficult. In this study, there were many limitations in terms of the conditions used for fiberoptic intubation, so the median intubation times for the NS (167 s) and INS (132.5 s) groups were longer than those in the above study. However, the median CPO intubation time (59 s) was shorter. Therefore, CPO can shorten the fiberoptic intubation time when the cervical spine is limited after anesthesia induction.

Therefore, CPO improves fiberoptic intubation efficiency by increasing the success rate and shortening the intubation time. We identified several possible causes for the fiberoptic intubation efficiency of CPO. First, the collapse of the tongue root and soft palate after anesthesia induction is reduced when oxygen is continuously supplied directly to the front end of the fiberoptic at a flow rate of 5 L/min [22]. Parke et al. [23] demonstrated that a low level of positive pressure was generated with nasal high flow. Thus, local positive pressure is produced in the oropharyngeal cavity as a result of the local impact on tissues in the pharyngeal cavity, which is similar to nasal high flow. Therefore, the cavity expands to a more appropriate volume, which improves tissue structure recognition, reduces intubation difficulty, increases the success rate of fiberoptic nasal intubation, and shortens the time needed for intubation. In addition, the use of negative pressure to clear secretions from the oropharyngeal cavity increases the difficulty of the procedure since negative pressure may further reduce the volume of the cavity by aggravating the collapse of the tongue root and the soft palate, making it more difficult to identify the tissue structures in the cavity.

Second, oropharyngeal secretions interfere greatly with fiberoptic nasal intubation [24]. We found during the experiment that the forward physical force produced by CPO could effectively clear secretions from the field of view, which was conducive to fiberoptic bronchoscopic guidance. Therefore, the rate of fiberoptic objective lens contamination was lower in the CPO group.

Finally, the long-term use of dry oxygen leads to the loss of airway mucosal moisture [11]; thus, CPO can be used to remove moisture, reducing the impact of secretions on the objective lens of the fiberoptic and thus making the view clearer during intubation. In this study, the longest duration of dry oxygen administration with continuous positive pressure was 181 s, and the patient was not harmed by this exposure.

A higher intubation success rate, shorter intubation time, and lower rate of contamination by secretions increase the efficiency of intubation and reduce the deoxidation time. CPO can also improve patient safety by improving oxygenation and reducing the number of intubations.

Hypoxia is a common complication of fiberoptic intubation [4]. Oxygen supplementation is an important step in fiberoptic intubation for difficult airways [6,10]. Studies have shown that it is beneficial to administer oxygen during intubation to increase the time to desaturation [25] or decrease the incidence of desaturation [26] during emergency intubation. In the past, some scholars have reported that oxygen administration via the Portex Swivel Adaptor during fiberoptic intubation can improve patient safety [27].

At present, there are different ways to supplement oxygen, such as low-flow oxygen, high-flow oxygen, and even jet ventilators, via supraglottic oxygenation in difficult airway patients. Badiger et al. [13] reported that high-flow oxygen inhalation through the nasal cavity reduces the risk of oxygen saturation decline among patients who are awake for endotracheal intubation and may optimize the conditions for intubation. However, patients need to pay for additional materials. Roh et al. [15] reported that continuous oxygen administration through the negative-pressure oxygen pathway can improve the success rate of fiberoptic intubation and reduce the influence of oral secretions on fiberoptic intubation. In this study, oxygen supplied through the negative-pressure suction channel was delivered directly to the front end of the fiberoptic device to fill the nasal cavity, laryngeal cavity, and other cavities and provide oxygen reserves for patients. When the glottis is exposed, oxygen directly enters the main trachea at a high speed and may even enter the terminal trachea to provide oxygen directly to patients. This method may increase oxygen reserves. Therefore, in our study, CPO improved SPO2 during fiberoptic intubation in patients with simulated cervical fractures. However, for pairwise comparisons, there was no significant difference, and a larger sample size is needed.

Multiple intubation attempts are another common complication of fiberoptic bronchoscopic intubation [4]. In our study, multiple intubations were also the main cause of intubation failure. Repeated intubation attempts may traumatize the airway and even make subsequent ventilation impossible. Therefore, fiberoptic intubation should not be performed more than three times, and help should be requested for the fourth time [6,10]. In our study, the number of intubations was limited, and CPO reduced the number of intubation attempts.

In this study, the pharyngeal mucosa of one patient was contacted during the removal of oral secretions by negative-pressure suction, resulting in pharyngeal mucosal bleeding, which prolonged the time required for fiberoptic nasal intubation. Therefore, CPO did not increase the incidence of intubation complications compared with NS and INS.

Earlier studies did not recommend continuous oxygen administration with fiberoptic agents [28], which may allow oxygen to enter the stomach, increase the risk of nausea and vomiting, and may lead to pulmonary barotrauma in patients with narrow airways. Flows of 35 L/min with mouth closure have been shown to create positive expiratory nasopharynx pressures of up to 5.3 cm H2O [25], and a pressure lower than approximately 20 cm H2O is required to open the esophagus closed by a sphincter. Our study did not measure the pressure in the oropharyngeal cavity caused by CPO, and further research is needed.

Our study has several limitations. This was a single-center study with a small sample size. It is necessary to further test the advantages of CPO in the intubation of fiberoptic devices in clinical practice. The oxygen supply rate was 5 L/min in this study. We did not further compare the effects of different flow rates of oxygen on fiberoptic intubation. We did not measure the specific pressure generated by continuous positive pressure of 5 L/min oxygen delivered through the negative-pressure suction pathway. We performed fiberoptic intubation in simulated cervical fracture patients, and we cannot determine whether this method of intubation actually prevented iatrogenic neurologic injury.

## Conclusions

In this study, we performed fiberoptic intubation in a difficult airway that simulated cervical spine trauma restricting cervical spine movement after anesthesia induction to avoid iatrogenic nerve injury caused by tracheal intubation. CPO through the negative-pressure suction pathway improves the fiberoptic intubation efficiency by increasing the success rate and shortening the operative time. In addition, the amount of time that patients may be hypoxic may decrease. The safety of fiberoptic intubation can be improved by increasing the oxygen supply during intubation and reducing the number of intubation attempts, hence decreasing intubation complications. Perhaps, CPO through the negative-pressure suction pathway is more suitable for fiberoptic intubation after anesthesia induction in difficult airway patients with limited cervical spine movement such as cervical spine fractures.
